# Understanding Prebiotic Allergy: An Evaluation of Basophil Activation Induced by Galacto‐Oligosaccharides

**DOI:** 10.1002/clt2.70150

**Published:** 2026-03-05

**Authors:** Si Yuan Leow, Hongmei Wen, Jian Yi Soh, Wen Chin Chiang, Youjia Zhong, Elizabeth Huiwen Tham, Wenyin Loh, Si Hui Goh, Dianne J. Delsing, Bee Wah Lee, Chiung‐Hui Huang

**Affiliations:** ^1^ Department of Paediatrics Yong Loo Lin School of Medicine National University of Singapore Singapore Singapore; ^2^ Khoo Teck Puat‐National University Children's Medical Institute National University Health System Singapore Singapore; ^3^ Department of Paediatrics Kandang Kerbau Women's and Children's Hospital Singapore Singapore; ^4^ FrieslandCampina Amersfoort the Netherlands

**Keywords:** basophil activation, carbohydrate allergy, galacto‐oligosaccharides

To the Editor,

Galacto‐oligosaccharides (GOS) are composed of one to seven galactose units linked to a glucose unit. They are enzymatically produced prebiotics supplemented widely in commercial infant milk formulas and dairy beverages for their health benefits. However, cases of allergic or anaphylactic reactions to GOS have been limited to Southeast Asian countries, including Singapore [1, 2], suggesting potential regional predisposing factors like genetic or environmental cofactors [3, 4]. In Singapore, *Blomia tropicalis* (*Blo t*), one of the most prevalent house dust mite species predominantly found in tropical and subtropical regions, is the putative primary sensitizer for GOS allergy [3]. Unlike conventional protein or glycoprotein allergens, GOS allergens are carbohydrates with molecular weight less than 2 kDa [5]. In this study, we review our current understanding and provide new findings to address how GOS trigger basophil activation.

GOS allergy presents as a typical IgE‐mediated food allergy, characterised by the rapid onset of type I hypersensitivity symptoms, following ingestion of GOS‐supplemented products [1, 2]. GOS‐allergic patients demonstrate IgE sensitization to GOS, with positive skin prick tests and detectable GOS‐specific IgE in plasma [3, 6]. Moreover, indirect basophil activation tests (iBAT) revealed that wortmannin, a phosphatidylinositol‐3‐kinase inhibitor, diminished GOS‐induced basophil activation in donor basophils passively sensitized with plasma from GOS‐allergic subjects [1]. To confirm that GOS triggers basophil activation via IgE binding to FcεRI, we present here results of iBAT using omalizumab, an anti‐IgE monoclonal antibody that blocks the binding of IgE to Fcε receptor. Basophils from atopic subjects without GOS allergy were stripped of surface IgE and passively sensitized with GOS‐specific IgE from GOS‐allergic patients' plasma, with or without preincubation with omalizumab. GOS‐induced basophil activation was observed in basophils re‐sensitized with GOS‐allergic patient's plasma, but not in basophils that resensitization with omalizumab treated plasma (Figure S1). Collectively, these findings substantiate the notion that GOS allergy is IgE‐mediated.

The mechanism by which low molecular weight GOS cross‐links IgE and activates basophils remains unclear. Low molecular weight allergens such as penicillin require binding to carrier proteins in plasma to activate basophils [7]. Our previous studies explored whether carrier proteins in serum or cell surface proteins may facilitate basophil activation. We showed that GOS activates basophils independent of serum proteins [1], and galectins (lectins that bind *β*‐galactoside sugars) [5].

To evaluate whether other cellular membrane proteins are involved in GOS‐induced basophil activation, we recruited three subjects with positive SPT and basophil activation test responses to GOS and *Blo t*. Of these, two were allergic to GOS (S1‐S2), however one declined the oral challenge (S3). Three subjects sensitized to *Blo t* but not to GOS were recruited as controls (C1‐C3). Purified basophils, identified as SSC^low^CD45^+^IgE^high^ cells by flow cytometry, with purity of 90.7% ± 3.36% were used in time‐lapse confocal microscopy to examine if GOS activated basophils without contact with other immune cells, including basophils and platelets. Additional information about subjects (Table S1 and Figure S2), study methodology and findings are available in the online repository.

To monitor basophil activation, purified basophils were stained with anti‐CD63 antibody and sulforhodamine conjugated avidin (Av.SRho). The latter detects secretory granules released during granule exteriorization [8]. Degranulated basophils were identified by both Av.SRho and CD63 staining. Upon stimulation with either *Blo t* (positive control allergen) or anti‐IgE, basophil activation was observed in all subjects (GOS allergic and controls) with similar percentages of degranulated basophils (Figure 1A). When purified basophils were stimulated with 1 mg/mL of GOS, the percentages of degranulated cells for all three GOS‐allergic subjects were 29.0% (S1), 21.8% (S2) and 22.0% (S3) (Figure 1A). Among the degranulated basophils, 60.0%, 70.6%, and 75.0% of the cells for subjects S1, S2, and S3, respectively, had no contact with neighboring basophils prior to degranulation (Table S2). These data indicate that basophil activation could occur without basophil‐basophil interaction (Figure 1B). When comparing responses between allergens, GOS‐allergic subjects showed greater basophil degranulation with *Blo t* (80.0%–96.2%) than GOS (21.8%–29.0%), suggesting that GOS is less potent than the multivalent protein allergen. Purified basophils of non‐GOS allergic atopic subjects did not respond to GOS stimulation. Consistent results were obtained with repeated experiments for GOS subjects.

**FIGURE 1:**
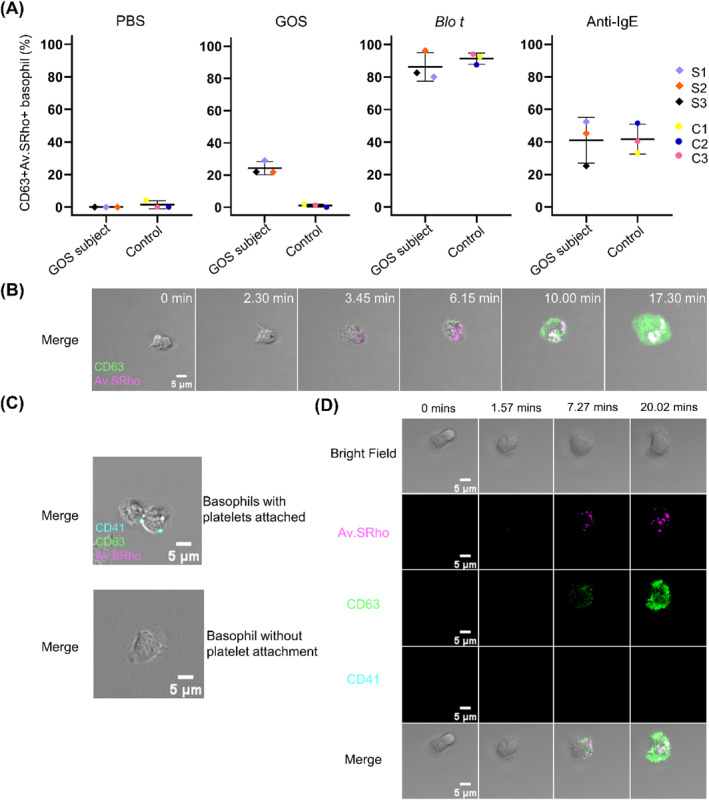
Time‐lapse confocal microscopy monitoring of basophil degranulation following GOS stimulation. (A) The percentage of degranulated basophils (CD63^+^Av.SRho^+^) in GOS allergic (S) (*n* = 3) and control (C) (*n* = 3) subjects at *T* = 30 min following stimulation with PBS, GOS, *Blo t*, or anti‐IgE. (B) Images of a GOS‐activated basophil. FITC anti‐CD63 antibody (green). Av.SRho (magenta). Representative of three GOS‐allergic subjects. Scale bar, 5 μm. (C) Purified basophils with or without platelet (cyan) attachment. Scale bar, 5 μm. (D) Images of a GOS‐activated basophil without platelet, from Video S1 (Online repository). Scale bar, 5 μm. All data were acquired on FV3000 Olympus confocal microscope.

As platelets can attach to basophils [9], experiments to exclude platelets involvement in GOS‐induced basophil activation were also made by concurrent platelet detection using anti‐CD41 (Figure 1C). Twelve out of seventeen activated basophils (70.6%) showed CD63 and Av.SRho signals without co‐expression of CD41 across all three GOS subjects (Figure 1D and Table S3), indicating that GOS‐induced basophil activation did not require platelet participation.

Our findings demonstrate that GOS can activate basophils at single‐cell level, without cell‐to‐cell contact, indicating that prior binding of GOS to membrane proteins on other cells is unnecessary. However, the limitation of this study is the requirement of a bovine serum albumin (BSA) supplemented medium, which was essential for cell stability. Without BSA, the CD63 expression was significantly reduced even with anti‐IgE stimulation (data not shown). Hence, BSA may have compensated for the absence of cell membrane proteins, thereby mediating IgE cross‐linking, which requires further investigation. Nevertheless, our findings suggest that GOS may directly cross‐link IgE, but such cross‐linking by such small carbohydrates is sterically difficult to explain. In Singapore, the estimated prevalence of GOS allergy is 3.5% among the atopic population (aged 5–60) [6]. However, the rarity of GOS allergy due to limited number GOS supplemented products here, constrained our sample size. Nonetheless, our results were consistent between patients and on repeated occasions (Tables S2 and S3). Future research using techniques like electron microscopy to identify IgE epitopes of GOS is essential to decipher exactly how small molecular allergens in GOS cross‐link IgE on basophils.

## Author Contributions

**Si Yuan Leow:** conceptualization, methodology, investigation, validation, formal analysis, visualization, writing – original draft, writing – review and editing. **Hongmei Wen:** investigation. **Jian Yi Soh:** writing – review and editing, resources. **Wen Chin Chiang:** writing – review and editing, resources. **Youjia Zhong:** writing – review and editing, resources. **Elizabeth Huiwen Tham:** writing – review and editing, resources. **Wenyin Loh:** writing – review and editing, resources. **Si Hui Goh:** writing – review and editing, resources. **Dianne J: Delsing:** writing – review and editing, resources. **Bee Wah Lee:** conceptualization, methodology, supervision, funding acquisition, project administration, writing – review and editing, writing – original draft, resources, formal analysis. **Chiung‐Hui Huang:** writing – review and editing, writing – original draft, project administration, resources, funding acquisition, supervision, conceptualization, methodology, formal analysis.

## Funding

This work was supported by the National Medical Research Council, Singapore [NMRC/CIRG/1487/2018 and MOH‐001702‐00].

## Ethics Statement

This study was reviewed and approved by Institutional Review Board of the National Healthcare Group, Singapore (ref: 2019/00571 and ref: 2013/00109). Informed written consents were obtained from adult subjects or child subject's legal guardian.

## Conflicts of Interest

Dianne J. Delsing is employed by FrieslandCampina, Amersfoort, The Netherlands. The rest of the authors declare that they have no relevant conflicts of interest.

## Supporting information


Supporting Information S1

Supporting Information S2

**Figure S1**: Omalizumab abolished the GOS‐induced basophil activation in indirect BAT assay. Buffy coat from control samples (C3‐C5) were subjected to acid stripping to remove membrane bound IgE on basophils. The cells were then incubated with plasma from GOS‐allergic subjects' (S4 and S5) or with plasma preincubated with omalizumab. Cells were subsequently stimulated with increasing concentration of GOS and the percentage of CD63+ cells among basophils was determined by flow cytometry.

**Figure S2**: GOS and Blo t‐induced basophil activation in GOS allergic and control subjects. Whole blood from GOS allergic subject (S1 to S3) (A) and control subjects (C1 to C3) (B) was stimulated with increasing concentration of GOS (ranges from 0.3 μg/mL to 1000 μg/mL) or Blo t (ranges from 10 ng/mL to 1000 ng/mL). The percentage of CD63+ cells among basophils was determined by flow cytometry.

**Table S1**: Clinical profiles of subjects.

**Table S2**: Results of basophil activation in GOS‐allergic and control subjects measured using time‐lapse confocal microscopy.

**Table S3**: Number of degranulated basophils without contact with neighbouring basophils and platelets in GOS‐allergic subjects measured using time‐lapse confocal microscopy.

**Video S1**: GOS‐induced basophil degranulation without neighbouring or platelet interaction. Purified basophils from GOS allergic subjects were stimulated with 1 mg/mL GOS at T=0 min. Purified basophils were stained with anti‐CD63 mAbs (green) and Av.SRho (magenta). Platelets were detected using anti‐CD41 mAbs (cyan). The video shown was recorded every 23 seconds for 20 minutes using time‐lapse confocal microscopy and processed using ImageJ. Data shown is from one representative basophil from a GOS allergic subject. Scale bar, 5μm. Data were acquired on a FV3000 Olympus confocal microscope.

## Data Availability

The data that support the findings of this study are available from the corresponding author upon reasonable request.
