# Supplementary material for: Understanding Prebiotic Allergy: An Evaluation of Basophil Activation Induced by Galacto‐Oligosaccharides
Source: Clin Transl Allergy. 2026 Mar 5;16(3):e70150. doi: 10.1002/clt2.70150 (PMC12962392; doi:10.1002/clt2.70150)
Supplement: Supplementary file 1 — Supporting Information S1 [file CLT2-16-e70150-s005.docx]

**Supplementary**

**Reagents**

The monoclonal antibodies (mAbs) used in this study were brilliant violet 421 (BV421)-labelled anti-human CD41 (HIP8; BioLegend, San Diego, CA), allophycocyanin (APC)-labelled anti-human CD45 (2D1, BioLegend, San Diego, CA), fluorescein isothiocyanate (FITC)-labelled anti-human CD63 (H5C6; BioLegend, San Diego, CA), phycoerythrin (PE)-labelled anti-human IgE (Ige21; eBioscience, Carlsbad, CA), RealBlue 705 (RB705)-labelled anti-human CD123 (6H6, BD Biosciences, Franklin Lakes, NJ) and anti-human IgE (G7-18, BD Biosciences, Franklin Lakes, NJ). Avidin-Sulforhodamine 101 conjugate (Av.SRho) was purchased from Abcam. Vivinal^®^ GOS syrup (GOS) was obtained from FrieslandCampina, The Netherlands. *Blomia tropicalis* (*Blo t*) extracts were prepared using in-house cultured *Blo t* mites [1].

**Subjects**

This study involved atopic subjects with history of rhinitis, eczema, wheezing/ asthma, and/ or food allergy. Five GOS sensitized subjects (S1-5) who are allergic to GOS and/or have positive BAT responses to GOS were enrolled. Another five subjects sensitized to *Blo t* but not to GOS were recruited as controls (C1-5). SPTs to GOS or *Blo t* were conducted with 7.2 mg/mL of GOS and 0.5 mg/mL of *Blo t* crude extracts*,* with histamine and saline as positive and negative controls, respectively. The SPT wheal size was recorded at 15 minutes, and wheal diameter of ≥3 mm was considered a positive SPT. The BAT to GOS was considered positive when the CD63 expression exceeded the cut-off value established previously [1]. Three GOS subjects (S1, S4, and S5) had a clinical history of GOS allergy, another (S2) had a positive oral GOS challenge, and one subject (S3) had a positive BAT to GOS but declined the oral challenge. The clinical profiles of subjects were summarized in Table S1. Informed written consents were obtained from adult subjects or parents of child subjects.

**Basophil activation test (BAT)**

BAT to *Blo t*, or GOS were conducted using sodium heparinized blood from GOS allergic and control subjects, as described previously [2,3]. Briefly, 100 µl of heparinized blood from subjects were incubated at 37 °C for 5 minutes before stimulation with increasing concentration of GOS (0.3 µg/mL to 1000 µg/mL) or *Blo t* (10 ng/mL to 1000 ng/mL) for 15 minutes. Cells were then washed in PBS/ EDTA and stained with PE-labelled anti-human IgE, and FITC-labelled anti-human CD63. Samples were then washed with 1% BSA/ PBS, subjected to erythrocyte lysis and fixed with 2% paraformaldehyde. Basophil activation was analysed by using flow cytometry and basophils were identified based on the side-scatter characteristics (SSC^low^) and IgE^high^ expression cells. BAT assay results were expressed as percentage of CD63^+^ cells among IgE^high^ cells.

**Indirect Basophil Activation Test (iBAT)**

The iBAT to GOS were performed following the protocol of Ruinemans-Koerts and Schmidt-Hieltjes with some modifications [4]. Briefly, buffy coats were collected from sodium heparinised blood of atopic subjects sensitized to *Blo t* and washed with 0.9% NaCl. A total of 3 ×10^7^ leucocytes were treated with 2 mL of cold stripping buffer (0.15 mol/L sodium dihydrogen phosphate monohydrate, 0.005 mol/L potassium chloride, pH 3.55) for 100 seconds. The cells were washed with 2 mL of basophil stimulation buffer (BSB) containing calcium, heparin, and IL-3 (Bühlmann, Basel, Switzerland) and resuspended in 1.5 mL of BSB. Cells were passively sensitized with IgE from GOS allergic patients (S4 and S5) by incubating 500 µl of IgE-stripped donor buffy coat cells in BSB with 130 µL of previously stored GOS patient plasma for 2 hours at 37 °C, with gentle mixing every 20 minutes. For omalizumab inhibition assays, patients' plasma was pre-incubated with 10 µg/mL of omalizumab (XOLAIR^®^) for at least 30 minutes before addition to donor cells. Basophils from donor C3 were re-sensitized with S4 plasma while C4 and C5 were re-sensitized with S5 plasma.

Cells were washed with Dulbecco’s PBS (DPBS), and resuspended in DPBS containing human IL-3 (Miltenyi Biotec). Basophil activation was performed as described in the BAT protocol with minor changes. Cells were pre-warmed at 37 °C for 10 minutes. GOS (10 µg/mL to 1000 ug/mL) diluted in DPBS were added and incubated for 15 minutes at 37 °C. Cells were stained with RB705-labeled anti-human CD123, PE-labeled anti-human IgE, and FITC-labeled anti-human CD63. Basophil activation was analyzed via flow cytometry using a FACSCanto II (BD Biosciences) and expressed as the percentage of CD63^+^ cells among SSC^low^CD123^high^IgE^+^ basophils.

**Time-lapse monitoring of basophil degranulation using confocal imaging**

Peripheral blood mononuclear cells (PBMCs) were first isolated from heparinized blood of GOS allergic and control subjects using Ficoll-Paque density gradient centrifugation. Basophils were then purified from PBMCs using the basophil isolation kit II (Miltenyi Biotec, Germany) according to the manufacturer’s instructions. The purity of basophils isolated was analysed using flow cytometry (CD45^+^IgE^high^) and was greater than 86% (Mean purity= 90.7%, SD=3.36%).

Confocal time-lapse monitoring of basophil degranulation was conducted according to the protocol described by Joulia et al. with slight modifications [5]. A total of 1x10^4^ basophils purified from GOS allergic and control subjects resuspend in Tyrode’s buffer (10 mM HEPES buffer (pH 7.4), 130 mM NaCl, 5 mM KCl, 1 mM MgCl_2_, 1.4 mM CaCl_2_, 5.6 mM glucose, and 1% BSA) supplemented with 4 ng/mL human IL-3 (Miltenyi Biotec) were added to Poly-D-lysine (Gibco) coated Nunc-Tek Lab chambered coverglass (Nunc, Roskilde, Denmark). Cells were warmed at 37 °C for 30 minutes. Prior to stimulating basophils with 2 µg/mL anti-human IgE, 1 mg/mL GOS, or 0.5 mg/mL *Blo t* at time T=0 min, 8 µg/mL Av.SRho and FITC-labelled anti-CD63 were added. Confocal laser scanning microscopy images were obtained with the FV3000 Olympus confocal microscope using the multi-area time-lapse function set at 40 x magnification, covering an area of 923 µm x 923 µm. Images were captured every 75 seconds for a total of 30 minutes. In this assay, 1 × 10⁴ basophils were seeded per well to minimize contact between neighbouring basophils. Imaging at 40x magnification provided sufficient high resolution for detailed assessment of basophil activation, but this limited the field of view to about 10 basophils per frame. To overcome this, multi-area time-lapse imaging (923 µm x 923 µm) was implemented, increasing the number of observed cells to approximate 80 cells per acquisition while maintaining a short imaging interval (75 seconds). Experiments were repeated for S1 and S2.

Degranulated basophils were identified by the presence of both Av.SRho and CD63 on the cell surface, alongside morphological characteristics such as cell elongation and spreading. Av.SRho is used to monitor basophil degranulation by staining the negatively charged proteoglycans of exteriorized granules [5]. The percentage of degranulated basophils (%) = number of degranulated basophils/ total number of basophils x 100%.

Degranulated basophils lacking cell contact were identified as basophils that had not been in contact with adjacent basophils prior to activation. The percentage of degranulated basophils without basophil-basophil contact (%) = number of degranulated basophils without cell contact prior to activation/ total number of degranulated basophils x 100%.

To study whether platelets contribute to GOS-induced basophil degranulated, sodium heparinised blood samples were collected from the same GOS subjects (S1-S3) at a different time point. Anti-CD41 mAbs was added in addition to Av.SRho and anti-CD63 mAbs. Purified basophils from GOS allergic subjects (n=3) were stimulated with 1mg/mL GOS at time T=0 min. With the addition of a fluorescent marker, BV421-labelled anti-human CD41, a longer scan time was required. To reduce the time interval between the images taken, a smaller imaging area was captured (318 µm x 636 µm) resulting in fewer cells captured per imaging. Images were taken approximate every 23 seconds for a total of 20 minutes.

**Statistical analysis**

Mann-Whitney U tests were used for comparing the percentage of basophil degranulation between the two groups (Prism8, GraphPad software). P-values of <0.05 were considered to be statistically significant. Confocal images shown were representative of repeated experiments and processed using the ImageJ (Fiji) software [6]. ImageJ software was also used for cell counting.

**Supplementary reference:**

1. Lee L, Zhong Y, Leow SY, et al. Allergy to prebiotic galacto-oligosaccharides: House dust mites-the putative primary sensitizer. *J Allergy Clin Immunol*. 2020;145(2):707-710.e5.

2. Chiang WC, Huang CH, Llanora GV, et al. Anaphylaxis to cow's milk formula containing short-chain galacto-oligosaccharide. *J Allergy Clin Immunol*. 2012;130(6):1361-7.

3. Lee L, Leow SY, Wen H, et al. An Evaluation of the Mechanisms of Galacto-Oligosaccharide (GOS)-Induced IgE Cross-Linking on Basophils in GOS Allergy. *Front Allergy*. 2022;3:840454.

4. Ruinemans-Koerts J, Schmidt-Hieltjes Y. Indirect Basophil Activation Test for Peanut Allergy Diagnosis Using Human Donor Basophils. *Methods Mol Biol*. 2024;2717:221-226.

5. Joulia R, Mailhol C, Valitutti S, Didier A, Espinosa E. Direct monitoring of basophil degranulation by using avidin-based probes. *J Allergy Clin Immunol*. 2017;140(4):1159-1162.e6.

6. Schindelin J, Arganda-Carreras I, Frise E, et al. Fiji: an open-source platform for biological-image analysis. *Nat Methods*. 2012;9(7):676-82.
