# Supplementary material for: Understanding Prebiotic Allergy: An Evaluation of Basophil Activation Induced by Galacto‐Oligosaccharides
Source: Clin Transl Allergy. 2026 Mar 5;16(3):e70150. doi: 10.1002/clt2.70150 (PMC12962392; doi:10.1002/clt2.70150)
Supplement: Supplementary file 2 — Supporting Information S2 [file CLT2-16-e70150-s002.pdf]

Supplementary Video S1.

GOS-induced basophil degranulation without neighbouring or platelet interaction. Purified basophils from GOS allergic subjects were stimulated with 1 mg/mL GOS at T=0 min. Purified basophils were stained with anti-CD63 mAbs (green) and Av.SRho (magenta). Platelets were detected using anti-CD41 mAbs (cyan). The video shown was recorded every 23 seconds for 20 minutes using time-lapse confocal microscopy and processed using ImageJ. Data shown is from one representative basophil from a GOS allergic subject. Scale bar, 5µm. Data were acquired on a FV3000 Olympus confocal microscope.
