# Supplementary material for: Understanding Prebiotic Allergy: An Evaluation of Basophil Activation Induced by Galacto‐Oligosaccharides
Source: Clin Transl Allergy. 2026 Mar 5;16(3):e70150. doi: 10.1002/clt2.70150 (PMC12962392; doi:10.1002/clt2.70150)
Supplement: Supplementary file 3 — Figure S1: Omalizumab abolished the GOS‐induced basophil activation in indirect BAT assay. Buffy coat from control samples (C3‐C5) were subjected to acid stripping to remove membrane bound IgE on basophils. The cells were then incubated with plasma from GOS‐allergic subjects' (S4 and S5) or with plasma preincubated with omalizumab. Cells were subsequently stimulated with increasing concentration of GOS and the percentage of CD63+ cells among basophils was determined by flow cytometry. [file CLT2-16-e70150-s003.pdf]

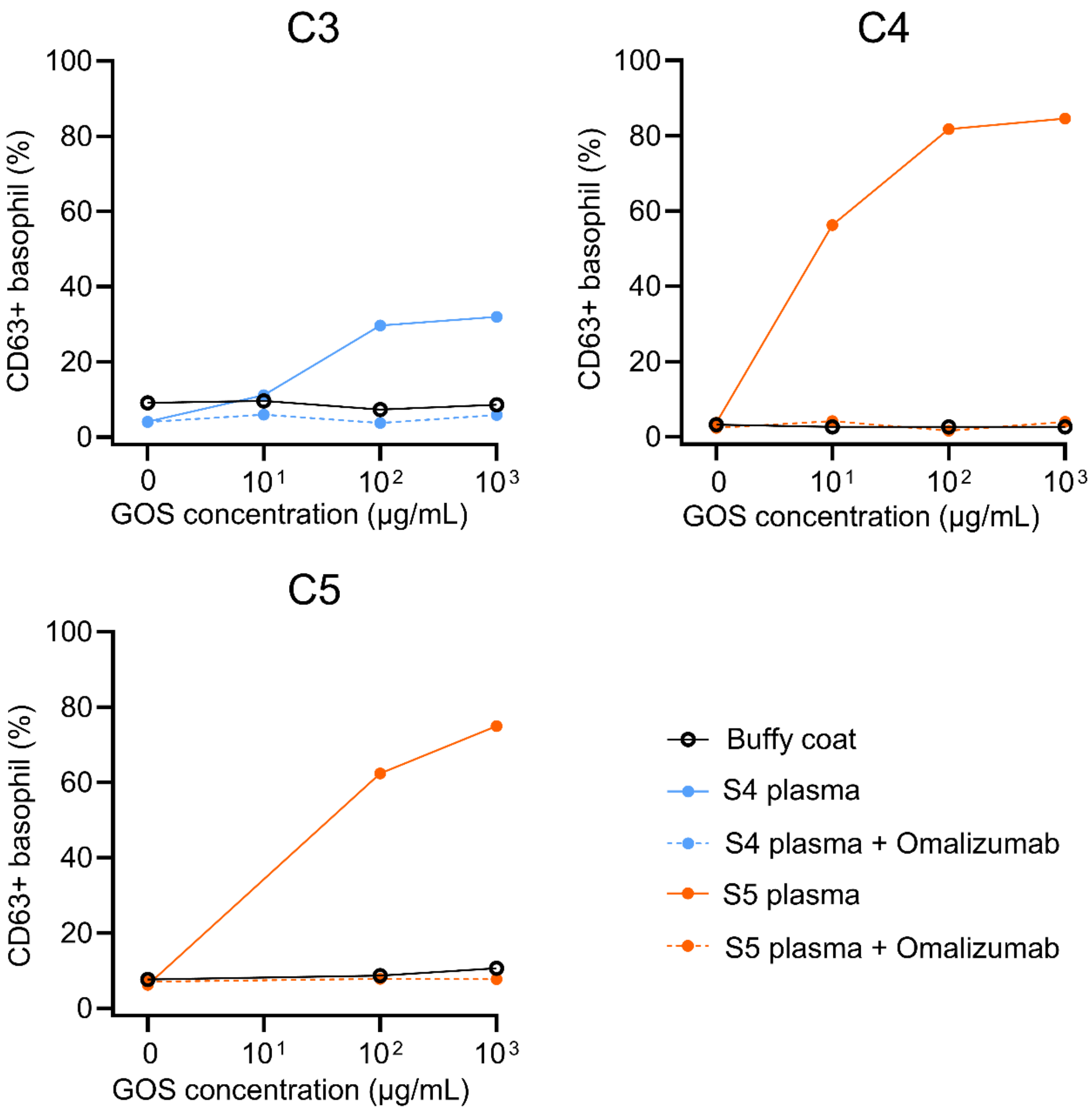

Supplementary Figure S1.

Omalizumab abolished the GOS-induced basophil activation in indirect BAT assay. Buffy coat from control samples (C3-C5) were subjected to acid stripping to remove membrane bound IgE on basophils. The cells were then incubated with plasma from GOS-allergic subjects' (S4 and S5) or with plasma preincubated with omalizumab. Cells were subsequently stimulated with increasing concentration of GOS and the percentage of $CD63^+$ cells among basophils was determined by flow cytometry.
