# Supplementary material for: Understanding Prebiotic Allergy: An Evaluation of Basophil Activation Induced by Galacto‐Oligosaccharides
Source: Clin Transl Allergy. 2026 Mar 5;16(3):e70150. doi: 10.1002/clt2.70150 (PMC12962392; doi:10.1002/clt2.70150)
Supplement: Supplementary file 4 — Figure S2: GOS and Blo t‐induced basophil activation in GOS allergic and control subjects. Whole blood from GOS allergic subject (S1 to S3) (A) and control subjects (C1 to C3) (B) was stimulated with increasing concentration of GOS (ranges from 0.3 μg/mL to 1000 μg/mL) or Blo t (ranges from 10 ng/mL to 1000 ng/mL). The percentage of CD63+ cells among basophils was determined by flow cytometry. [file CLT2-16-e70150-s004.pdf]

(A)

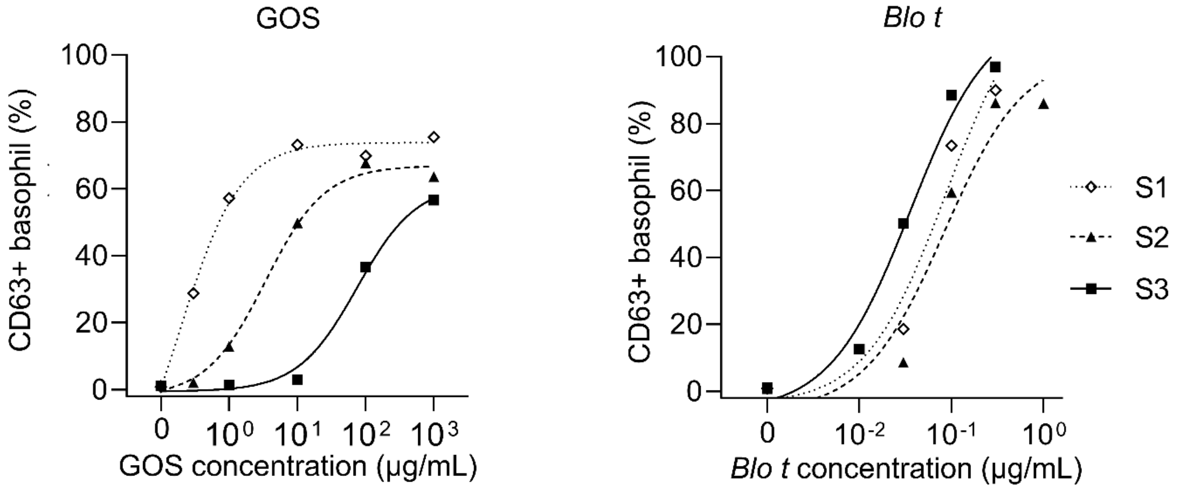

(B)

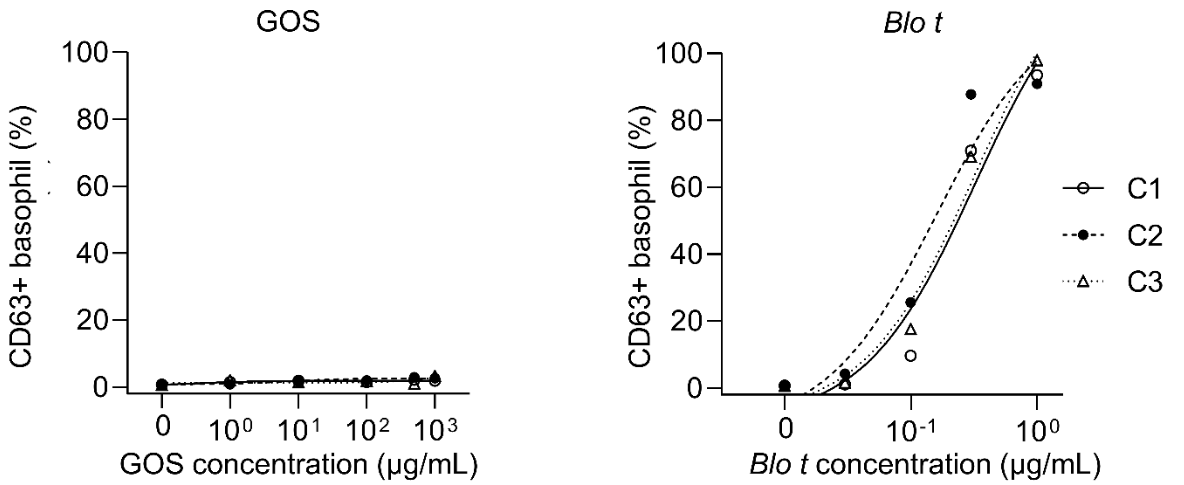

Supplementary Figure S2.

GOS and *Blo t*-induced basophil activation in GOS allergic and control subjects. Whole blood from GOS allergic subject (S1 to S3) **(A)** and control subjects (C1 to C3) **(B)** was stimulated with increasing concentration of GOS (ranges from 0.3 μg/mL to 1000 ug/mL) or *Blo t* (ranges from 10 ng/mL to 1000 ng/mL). The percentage of CD63+ cells among basophils was determined by flow cytometry.
