# Supplementary material for: Understanding Prebiotic Allergy: An Evaluation of Basophil Activation Induced by Galacto‐Oligosaccharides
Source: Clin Transl Allergy. 2026 Mar 5;16(3):e70150. doi: 10.1002/clt2.70150 (PMC12962392; doi:10.1002/clt2.70150)
Supplement: Supplementary file 5 — Table S1: Clinical profiles of subjects. [file CLT2-16-e70150-s008.pdf]

Supplementary Table S1. Clinical profiles of subjects

Subject ID.	Age of recruitment (Years)	Gender	Skin prick test (SPT): wheal size (mm)		History of allergic disorders	Clinical history of GOS allergy
			GOS	*Blo t*		
S1	17	Male	8x10	10x12	Rhinitis, Eczema, Wheezing/ Asthma, Food allergy	Acute anaphylaxis upon ingestion of GOS supplemented formula
S2	34	Male	5x5	10x10	Wheezing/ Asthma	Positive oral challenge to GOS
S3	39	Female	3x3	5x5	Rhinitis	Declined oral challenge to GOS
S4	6	Male	3x3	NA	Rhinitis, Eczema, Wheezing/ Asthma	Acute anaphylaxis upon ingestion of GOS supplemented formula
S5	7	Female	7x6	5x9	Rhinitis, Eczema	Acute anaphylaxis upon ingestion of GOS supplemented formula
C1	46	Female	0	6x8	Rhinitis, Eczema	NA
C2	34	Male	0	6x8	Rhinitis, Eczema, Food allergy	NA
C3	41	Female	0	10x9	Rhinitis, Eczema	NA
C4	32	Female	0	3x4	Rhinitis, Eczema	NA
C5	23	Female	0	5x5	Rhinitis	NA

GOS-allergic subjects: S1-S5; Control subjects:C1-C5.

S1-S3 and C1-C3 underwent time-lapse confocal microscopy to monitor basophil degranulation following GOS stimulation.

S4-S5 and C3-C5 participated in indirect basophil activation tests with omalizumab.

Not applicable; NA.
