# Supplementary material for: Understanding Prebiotic Allergy: An Evaluation of Basophil Activation Induced by Galacto‐Oligosaccharides
Source: Clin Transl Allergy. 2026 Mar 5;16(3):e70150. doi: 10.1002/clt2.70150 (PMC12962392; doi:10.1002/clt2.70150)
Supplement: Supplementary file 6 — Table S2: Results of basophil activation in GOS‐allergic and control subjects measured using time‐lapse confocal microscopy. [file CLT2-16-e70150-s007.pdf]

Supplementary Table S2. Results of basophil activation in GOS-allergic and control subjects measured using time-lapse confocal microscopy

Subject	Stimuli	Number of basophils	Percentage of degranulated basophils	Percentage of degranulated basophils without contact with neighbouring basophils
S1	Anti-IgE	88	52.3	
	Blo t	45	80.0	
	GOS	69 (76)	29.0 (31.6)	60.0 (50.0)
	PBS	70 (110)	0.0 (0.0)	
S2	Anti-IgE	53	45.3	
	Blo t	52	96.2	
	GOS	156 (48)	21.8 (29.2)	70.6 (71.4)
	PBS	66 (75)	0.0 (0.0)	
S3	Anti-IgE	79	25.3	
	Blo t	86	82.6	
	GOS	91	22.0	75.0
	PBS	72	0.0	
C1	Anti-IgE	72	33.3	
	Blo t	107	92.5	
	GOS	114	1.8	
	PBS	47	4.3	
C2	Anti-IgE	97	51.5	
	Blo t	80	87.5	
	GOS	102	0.0	
	PBS	85	0.0	
C3	Anti-IgE	104	40.4	
	Blo t	101	94.1	
	GOS	156	1.3	
	PBS	104	0.0	

GOS-allergic subjects: S1-S3; Control subjects:C1-C3

Values in parentheses were from repeated measurements on separate occasion; not shown in Figure 1A. Experiments were repeated for S1 and S2.

Degranulated basophils (%) = number of degranulated basophils/ total no of basophil x 100

Degranulated basophils without contact with neighbouring basophil (%) = number of degranulated basophils without contact/ total number of degranulated basophil x 100

Data were acquired on a FV3000 Olympus confocal microscope; imaging area captured: 923 µm x 923 µm.
