# Supplementary material for: Understanding Prebiotic Allergy: An Evaluation of Basophil Activation Induced by Galacto‐Oligosaccharides
Source: Clin Transl Allergy. 2026 Mar 5;16(3):e70150. doi: 10.1002/clt2.70150 (PMC12962392; doi:10.1002/clt2.70150)
Supplement: Supplementary file 7 — Table S3: Number of degranulated basophils without contact with neighbouring basophils and platelets in GOS‐allergic subjects measured using time‐lapse confocal microscopy. [file CLT2-16-e70150-s006.pdf]

Supplementary Table S3. Number of degranulated basophils without contact with neighbouring basophils and platelets in GOS-allergic subjects measured using time-lapse confocal microscopy

	**Number of basophils**	**Number of degranulated basophils**	**Number of degranulated basophils without contact with neighbouring basophils and platelets**
S1	15	9	7
S2	28	6	3
S3	14	2	2
Total	57	17	12

GOS-allergic subjects: S1-S3

Data were acquired on a FV3000 Olympus confocal microscope; imaging area captured: 318 µm x 636 µm.
